# Alternative Splicing in Plant Genes: A Means of Regulating the Environmental Fitness of Plants

**DOI:** 10.3390/ijms18020432

**Published:** 2017-02-20

**Authors:** Xudong Shang, Ying Cao, Ligeng Ma

**Affiliations:** College of Life Sciences and Beijing Key Laboratory of Plant Gene Resources and Biotechnology for Carbon Reduction and Environmental Improvement, Capital Normal University, Beijing 100048, China; 2160801011@cnu.edu.cn (X.S.); ying.cao@cnu.edu.cn (Y.C.)

**Keywords:** gene expression, alternative splicing, transcriptional regulation, environmental fitness, plant

## Abstract

Gene expression can be regulated through transcriptional and post-transcriptional mechanisms. Transcription in eukaryotes produces pre-mRNA molecules, which are processed and spliced post-transcriptionally to create translatable mRNAs. More than one mRNA may be produced from a single pre-mRNA by alternative splicing (AS); thus, AS serves to diversify an organism’s transcriptome and proteome. Previous studies of gene expression in plants have focused on the role of transcriptional regulation in response to environmental changes. However, recent data suggest that post-transcriptional regulation, especially AS, is necessary for plants to adapt to a changing environment. In this review, we summarize recent advances in our understanding of AS during plant development in response to environmental changes. We suggest that alternative gene splicing is a novel means of regulating the environmental fitness of plants.

## 1. Introduction

Nearly 90% of the protein-coding genes in plants are interrupted genes; that is, the coding region is divided by introns. Therefore, an essential step in gene expression is the removal of introns through the splicing of precursor mRNA transcripts (pre-mRNAs) [1,2]. Splicing is performed by the spliceosome, a large ribonucleoprotein (RNP) complex that assembles around splice sites in the introns of a pre-mRNA molecule and then removes the introns catalytically through sequential phosphodiester transfer reactions [3,4,5,6]. During splicing, 5′ and 3′ splice sites, which mark the beginning and end of each intron in a pre-mRNA, together with the branch site (a consensus sequence-containing region located near the 3′ splice site) are recognized by the uridine (U)-rich small nuclear RNPs (snRNPs) U1, U2, U4, U5, and U6, and a multitude of non-snRNP splicing factors, including U2AF65, U2AF35, and serine/arginine-rich (SR) proteins [7]. Together, these factors form one of the most complex machines in cells, the spliceosome, which performs the two transesterification reactions that are necessary to excise introns and join together the selected exons [8]. Spliceosome assembly at an intron is a highly ordered and dynamic process that is guided by consensus sequences [9].

Genome-wide transcriptome mapping has revealed that the extent of alternative splicing (AS) in plants ranges from 42% to 61% [10,11,12]. In AS, a single pre-mRNA can produce more than one mRNA through the use of alternative splice sites. AS is a regulated process that increases the diversity of an organism’s transcriptome and proteome. It can regulate transcript levels by producing new unstable mRNA isoforms, which can be degraded by nonsense-mediated decay (NMD) [13,14]. AS can also produce alternate functional mRNAs encoding protein isoforms with differences in subcellular localization, stability, or function by changing or completely removing functional domains via the introduction of a premature termination codon (PTC), intron retention, or alternative 3′ or 5′ splice site selection [15]. New isoforms of transcripts, proteins, or even polypeptides may act as dominant-negative inhibitors of the authentic proteins by means of interactions with dimerization partners [16]. Therefore, the precision and efficiency of alternative splicing are critical factors in gene function. Given their sessile nature, plants must adapt immediately to environmental changes to ensure their survival. Here, we summarize recent advances in our understanding of AS as it relates to the environmental fitness of plants.

## 2. Ambient Temperature and Vernalization-Mediated Flowering

Higher plants go through numerous developmental transitions during their life cycle. Among these transitions, the floral transition is the best studied. Analyses of the regulation of flowering in *Arabidopsis* have demonstrated how environmental signals and endogenous cues may be integrated to create a developmental switch in plants [17]. In recent decades, key regulators of flowering time and their targets have been described in *Arabidopsi*s.

To determine the right moment for flowering, plants utilize many environmental signals, including photoperiod (day length), light quality, biotic/abiotic stresses, and temperature. Temperature in particular has been shown to have tremendous effects on the timing of flowering; in the vernalization response of *Arabidopsis thaliana*, plants quantitatively sense long-term cold exposure and epigenetically store this information to regulate flowering time [18]. In contrast, our knowledge of the genetic and molecular mechanisms that regulate flowering time in response to small changes in ambient temperature (i.e., the thermosensory pathway) is limited. Evidence indicates that AS plays an important role in the ability of plants to measure ambient temperatures and to integrate this external information with endogenous signals to regulate flowering [19,20].

In *A. thaliana*, the central mechanism of accelerated flowering in response to prolonged cold exposure is repression of the transcription of *FLOWERING LOCUS C* (*FLC*), which encodes a MADS-box transcription factor and negative regulator of flowering. MADS-domain transcription factors have important roles in the development of plants throughout their life cycle, including flowering [21]. *FLC* expression is regulated by several pathways: the *FRIGIDA* pathway, which up-regulates *FLC* expression; the autonomous pathway, which down-regulates *FLC* expression; the vernalization pathway, a cold-induced epigenetically silenced *FLC* expression during winter [22]. *COOLAIR* is a non-coding RNA, which fully encompasses *FLC* in the antisense direction [23]. The level of *FLC* expression is associated with the level of *COOLAIR* splicing isoforms. Class I *COOLAIR* variants, which links with low level of *FLC* expression, uses a small intron and polyadenylation at a proximal region. Class II *COOLAIR* variants, linking with high level of *FLC* expression, is associated with a large intron and polyadenylation at a distal region [24,25]. PRP8 is a conserved and central component of the spliceosome, mutation in PRP8 influences the splicing efficiency of *COOLAIR* introns, which reduces *COOLAIR* proximal poly(A) site usage [25]. Furthermore, the splicing of *COOLAIR* plays an important role in the adaptive evolution of flowering time in *Arabidopsis thaliana* accessions. SNP259, a single natural intronic polymorphism located in *FLC*, can significantly change *COOLAIR* splicing, further affecting *FLC* expression and flowering time. SNP259 is a major contributor to the natural variation in *FLC* haplotypes [26]. In addition, long noncoding RNA not only acts as AS target, but is also involved in regulating AS. AS competitor long noncoding RNA (*ASCO*-lncRNA) can hijack nuclear speckle RNA-binding protein (NSR) to affect the splicing patterns of several NSR-regulated mRNA targets that specifically promote lateral root growth in *Arabidopsis* [27].

SHORT VEGETATIVE PHASE (SVP) and FLOWERING LOCUS M (FLM) also belong to MADS-box proteins. *FLM* is subject to temperature-dependent AS. Two splice variants, *FLM-β* and *FLM-δ*, which differ in the incorporation of either the second or third cassette exon and can be translated into two protein isoforms, have been detected in the Columbia-0 accession [28]. Interestingly, the ratio of *FLM-β* to *FLM-δ* changes in response to deviations in ambient temperature [29]. *FLM-β* is down-regulated in response to increases in temperature, suggesting that *FLM-β* and *FLM-δ* have different roles in the control of flowering by ambient temperature [30]. However, the biological functions of *FLM-β*/*δ* are unclear.

Recent discoveries have revealed a combinatorial role for *FLM* and *SVP* in flowering, with ambient temperature modulating both factors in different ways. *FLM-β* and *FLM-δ* encode proteins that interact antagonistically with SVP. FLM-β was demonstrated to bind DNA only in the presence of SVP in vitro; however, FLM-δ could not bind DNA in vitro, thus, it prevented SVP from binding the target DNA in a dose-dependent manner [19,20]. These results indicate that FLM-β and -δ protein levels mirror those of their corresponding mRNAs, which are regulated by ambient temperature changes. These results suggest a working model in which FLM-δ acts as a dominant-negative isoform of FLM that renders the SVP-FLM-β complex inactive, thereby indirectly promoting the flowering transition at elevated temperatures [19,20].

Similar to *FLM*/*MAF1*, the *MADS AFFECTING FLOWERING 2* (*MAF2*) gene, which is closely related to *FLM*, is subjected to temperature-dependent AS to produce two isoforms, var1 and var2. Only *MAF2* var1 encodes a functional full-length MIKC-type MADS-domain transcription factor that is sufficient to repress flowering when overexpressed in *A. thaliana* accession Ll-2, which does not express *MAF1–MAF4* [31,32]. Similar to *FLM*, the repressive isoform var1 is regulated by temperature-dependent AS. In addition, plants overexpressing *MAF2* var*2* are not early flowering, suggesting that var2 does not act as a dominant-negative protein, as is the case for FLM-δ.

**Figure 1:**
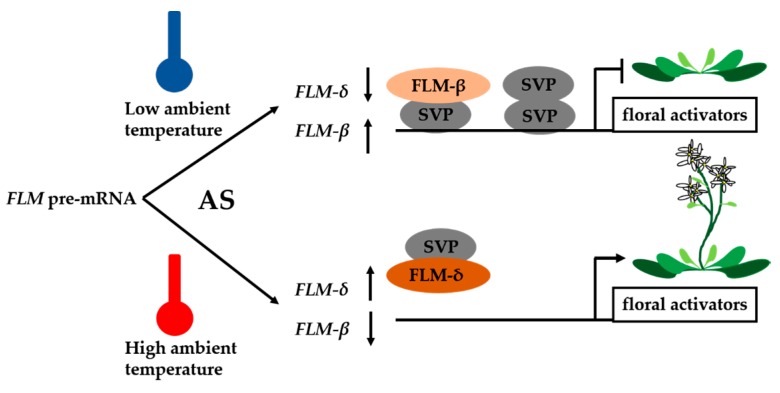
Model of the temperature-dependent *FLOWERING LOCUS M* (*FLM*) function. At low temperature, SHORT VEGETATIVE PHASE (SVP) can form homodimer or interact with FLM-β to form heterodimer to repress floral activators. When the temperature increased, the alternative splicing of *FLM* pre-mRNA increased the level of *FLM*-*δ*. FLM-δ proteins compete with the FLM-β to form FLM-δ-SVP complex and repress the transcription of floral activator genes. AS, alternative splicing.

In summary, AS functions as a “thermometer” in plants, measuring moderate changes in ambient temperature (Figure 1). Although the effects of different splice variants (e.g., *FLM-β*/*δ* and *MAF2* var1/2) on flowering are clear, the exact mechanism by which temperature-dependent AS modulates the onset of flowering is poorly understood. Furthermore, ambient temperature-responsive splicing factors that regulate AS have not yet been discovered.

## 3. The Circadian Clock in Plants

The circadian clock, an endogenous timekeeper that generates rhythms with an approximately 24 h period, plays critical roles in diverse aspects of plant growth and development, and coordination of biological processes with daily environmental cycles [33,34]. It was reported that the expression of about 30% of the genes in *Arabidopsis* genome is controlled under the circadian clock [35,36,37].

The circadian clock is composed of several interlocking regulatory feedback loops in *Arabidopsis* [38]. *CIRCADIAN CLOCK-ASSOCIATED1* (*CCA1*) and *LATE ELONGATED HYPOCOTYL* (*LHY*) repress the expression of *TIMING OF CAB EXPRESSION 1* (*TOC1*) through direct binding to its promoter [39,40]. Recent studies have shown that TOC1 binds directly to specific morning elements in the promoters of *CCA1* and *LHY* as a transcriptional repressor and suppresses CCA1/LHY accumulation [41,42,43]. In the morning loop, CCA1 and LHY directly activate the expression of *PSEUDO RESPONSE REGULATOR7* (*PRR7*) and *PRR9*, two homologs of *TOC1*; in turn, PRR7 and PRR9 suppress the expression of *CCA1* and *LHY* [44,45]. In the evening loop, TOC1 represses the expression of *GIGANTEA* (*GI*), while the expression of *TOC1* is up-regulated by GI [46]. The proteins encoded by *EARLY FLOWERING* (*ELF*)*3*, *ELF4*, and *LUX ARRHYTHMO* form the so-called evening complex (EC), which suppresses the expression of *TOC1*, *GI*, and *PRR9* [47,48]. Together, these interlocking central, morning, and evening loops form the basis of the circadian clock in *Arabidopsis*.

To date, most studies of the regulation of the circadian clock in both animals and plants have focused on the transcriptional and post-translational regulatory mechanisms. The roles of AS in the regulation of the circadian clock have been uncovered recently. Through whole-genome sequencing and RT-PCR analysis, two *CCA1* transcripts, *CCA1α* and *CCA1β*, have been reported [49,50]. *CCA1β*, an alternatively spliced variant of *CCA1* that retains the fourth intron, is conserved in the dicot tree *Populus* and the monocot grasses *Oryza* and *Brachypodium*, suggesting its functional importance; in addition, *CCA1β* is accumulated under high light conditions but decreased in cold conditions. The roles of these variants in regulating the circadian clock have been recently reported [50].

CCA1β has a dimerization domain, like CCA1α, but lacks the N-terminal DNA-binding MYB motif [50,51]. Homodimerization and heterodimerization of CCA1α and LHY are required for these proteins to regulate circadian rhythms [52]. The splice variant CCA1β, as a dominant regulator, represses CCA1α/LHY heterodimerization by competing with CCA1α and LHY to form nonfunctional CCA1α/CCA1β and CCA1β/LHY heterodimers, revealing the regulatory role of the AS of *CCA1* in circadian rhythms [50]. Recent research showed that SR45 bound specifically to the retained *CCA1* intron in vitro, suggesting that SR45 is involved in the regulation of intron splicing [53].

Protein arginine methyltransferase 5 (AtPRMT5) has been shown to regulate circadian period in *Arabidopsis*. PRMT5, a type II protein arginine methyltransferase, catalyzes the methylation of diverse non-histone proteins, including components of the spliceosome (e.g., AtSmD1 and AtLSm4); the reduction in the methylation levels of SmD1 and LSm4 causes the splicing defects in genes involved in multiple biological processes, including the circadian clock, probably by regulating 5′ splice site recognition [54,55]. The circadian period is lengthened by mutations in AtPRMT5 [56]. *Atprmt5* exhibits defects in the AS of *PRR7* and *PRR9*, resulting in lengthening of the circadian period; thus, AS regulates the circadian clock at the post-transcriptional level [54]. *LSM4* and *LSM5*, which encode core components of the spliceosomal U6 snRNP complex, can be methylated by PRMT5. *lsm4* and *lsm5* plants display a long period phenotype as well as aberrant splicing of several clock genes, including *CCA1* and *TOC1*, but not *PRR9*, suggesting that the effect of PRMT5 on the circadian clock is not simply due to its effect on LSM4.

Besides PRMT5, which indirectly affects the splicing of clock genes, the involvement of splicing factors in the circadian clock control has been demonstrated [57,58]. The Ski-interacting protein (SKIP) is the first splicing factor that is required for the regulation of circadian clock. SKIP is a conserved component of the precursor RNA processing (Prp)19 complex, a sub-complex of spliceosome complexes B and C, which are required for catalysis of the first and second steps in pre-mRNA splicing in yeast and human cells [59]. The mutation of *SKIP* has dramatic effects on the circadian clock in *Arabidopsis*. For example, the *skip-1* mutant exhibits a temperature-sensitive long period phenotype and changes in light input and clock sensitivity to resetting by light [57]. Consistent with the role of SKIP in mammals (SKIP) and yeast (Prp45), AtSKIP encodes a conserved SNW domain-containing protein. AtSKIP co-localizes in nuclear speckles with the spliceosome components U1-70k [60] and SR45 [61], and it associates with the pre-mRNAs of clock genes, including *PRR7* and *PRR9* [57,62]. Defects in the AS of *PRR7* and *PRR9* partially contribute to the lengthened period of the clock in the *skip-1* mutant [57,62].

Similarly, the RNA-binding protein SPLICEOSOME TIMEKEEPER LOCUS 1 (STIPL1) is homologous to the spliceosomal proteins Ntr1p in *Saccharomyces cerevisiae* and TFP11 in humans, which are required for spliceosome disassembly. The mutation of *STIPL1* increased levels of the intron-retained variants of *CCA1*, *LHY1*, *TOC1*, *PRR9*, and *GI*, and this may contribute to the observed clock phenotype [58], indicating the requirement of STIPL1 for the correct splicing of clock genes. The spliceosomal snRNP assembly factor GEM NUCLEAR ORGANELLE ASSOCIATED PROTEIN 2 (GEMIN2) plays an important role in modulating the effect of low temperatures on the splicing of certain pre-mRNAs (e.g., *TOC1* and *PRR*s), and it attenuates the effects of temperature on the period length of the circadian clock [63]. Recently, another regulator, SICKLE (SIC), a conserved proline/serine-rich protein found in nuclear foci, emerged as a link between the circadian clock, temperature compensation, and AS. The *sic-3* mutant exhibits defects in the clock genes, such as arrhythmic or low-amplitude expression of several core circadian clock genes under cool ambient temperature cycles, but no defect was observed under light-dark entrainment. Additionally, *sic* exhibits increased levels of splice variants of *LHY*, *ELF3*, *CCA1*, and *PRR7*. Further, compared to wild type, *sic* has a broader range of temperature conditions under which these splice variants occur, particularly at cool temperatures [64].

**Figure 2:**
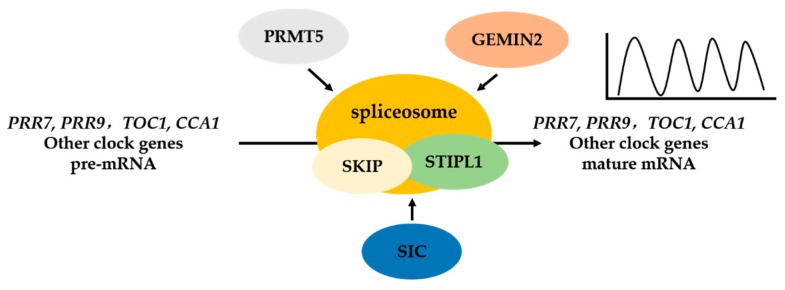
Components of spliceosome and splicing regulators mediate alternative splicing of the circadian clock genes in *Arabidopsis*. The Ski-interacting protein (SKIP), SPLICEOSOME TIMEKEEPER LOCUS 1 (STIPL1) are components of the spliceosome. Protein arginine methyltransferase 5 (PRMT5), GEM NUCLEAR ORGANELLE ASSOCIATED PROTEIN 2 (GEMIN2), SICKLE (SIC) are regulators of spliceosome. These splicing factors are involved in regulating the circadian clock through alternative splicing of clock genes pre-mRNAs, such as *PRR7*, *PRR9*, *TOC1*, *CCA1*. These studies suggest that pre-mRNA splicing, a post-transcriptional gene expression regulation, is a new means of circadian clock regulation.

The above mentioned results indicated that AS is essential for normal functioning of the circadian clock in *Arabidopsis* (Figure 2). The circadian oscillator plays an important role in the interaction between plants and their environment by synchronizing endogenous biological activities, biochemical processes, and behavior with daily environmental changes in the day-night cycle. Interlocking transcription-translation feedback loops form the basis for regulation of the clock. Recent studies also showed that AS is required for the regulation of the circadian clock in *Drosophila*, and *Neurospora*, suggesting that AS, a key step in post-transcriptional gene expression regulation, is a general mechanism of clock regulation. However, the detailed molecular mechanisms of AS that regulate the circadian clock, how the circadian clock regulates AS in response to external environment changes, and how endogenous developmental processes affect the circadian clock via AS are far from clear. The spliceosome has been described as a sea of proteins; indeed, more than 200 components have been purified from the mammalian spliceosome [65]. However, only a small proportion of them have been functionally characterized. Future studies should identify the roles of splicing factors or AS in the regulatory network of the circadian clock.

## 4. Abiotic Stress Responses

Given their sessile nature, plants are dependent on their immediate environment for survival, and the growth and development of plants are heavily affected by environmental cues [66,67,68]. AS in response to abiotic/biotic stresses has a wide range of effects on plants, and most genes involved in plant stress responses are said to be regulated by AS.

Heat shock (HS) transcription factors (Hsfs) are key regulators of the response of plants to heat stress; thus, HS-induced transcriptional regulation has been extensively studied [69]. Recently, AS has been shown to be critical for the HS-inducible expression of *HsfA2*. *HsfA2* contains a single 324-nucleotide intron that is fully spliced at 22 °C to generate the full-length *HsfA2* transcript [70]. Moderate heat (37 °C) activates a 31-nucleotide cryptic miniexon within the *HsfA2* intron to generate a splice variant *HsfA2-II*. *HsfA2-II* contains a PTC within the miniexon, thus is degraded by NMD. The third splice variant *HsfA2-III* is generated through the cryptic 5′ splice site in the intron, which is activated by extreme heat (42–45 °C), while *HsfA2-II* decreases [71]. *HsfA2-III* encodes a truncated protein, S-HsfA2, which localizes to the nucleus; it contains an Hsf helix-turn-helix DNA binding motif, and can bind to HS elements in the *HsfA2* promoter, generating a positive autoregulatory loop that controls HsfA2 expression through AS.

The maize gene *ZmrbohB* is required for the production of reactive oxygen species (ROS) in response to avirulent pathogens and several abiotic stresses. ROS levels need to be finely tuned to prevent toxicity, and to support their activity as signaling molecules [72,73]. *ZmrbohB* has two alternatively spliced isoforms, *ZmrbohB-δ* and *ZmrbohB*-*β* (which retains intron 11); these isoforms carry a PTC that probably leads to NMD in response to several abiotic stimuli, including cold, heat, ultraviolet radiation, and salt stress [74].

The splicing of some transcription factor genes also undergo AS under stressful conditions. For example, two splice variants of *OsDREB2B* are differentially expressed in response to heat and drought stresses in rice [75]. The transcript of *OsDREB2B1* is more abundant under normal growth conditions; it contains a 53-base pair exon 2 insertion in the mature mRNA that introduces an open reading frame shift. In plants exposed to high temperature stress, *OsDREB2B2*, in which exon 2 is spliced out in the mature mRNA, dominates. Through AS, rice can produce dehydration-responsive element-binding protein2 (DREB2B) rapidly, independentally of transcriptional activation. A similar mechanism of AS has been described for *WDREB2* in wheat, and for its orthologs *HvDRF1* in barley and *ZmDREB2A* in maize [76,77,78].

The splicing of non-protein coding RNA transcripts is an emerging area of study, specifically in plant systems. The first example of environmental changing-induced AS is from the expression of miR400. miR400 is located in the intron of a protein-coding gene. Heat stress-induced AS of a transcript that contains this intron results in decreased production of the mature miRNA by affecting miRNA processing. The altered miR400 level in turn changes the level of its host transcript [79].

A number of RNA processing factors is associated with plant responses to abscisic acid (ABA). RNA binding motif protein 25 (RBM25) binds to the last intron in *HAB1* pre-mRNA and regulates its AS to produce two splice variants: *HAB1.1* and *HAB1.2*. *HAB1.1* contains four exons and encodes a protein that interacts with and inhibits the kinase activity of SnRK2.6/OST1, switching ABA signaling off. *HAB1.2* contains four exons and the last intron; it encodes a truncated protein lacking 105 amino acids at the C-terminal end. HAB1.2 is able to interact with SnRK2/OST1, but it cannot inhibit the protein’s kinase activity; thus, ABA signaling remains on [80,81]. Therefore, the alternative splicing of *HAB1* pre-mRNA results in the production of two variants of *HAB1* mRNA, and translates to two functional antagonistic proteins in ABA signaling pathway. Thus, AS for genes encoding the components of ABA signaling pathway is critical for ABA function.

In addition to the induction of AS by environmental stress, evidence shows that spliceosomal proteins play crucial roles in the proper function of abiotic stress response pathways in *Arabidopsis*. SR proteins bind splicing signal sites and intronic and exonic splicing enhancer/silencer sequences through interactions with multicomponent splicing factors to determine splice site selection and where the spliceosome assembles. SR45 is an SR-like protein with two SR domains. The mutation of SR45 in *Arabidopsis* produces pleiotropic developmental defects, including altered leaf and flower morphology, delayed root growth, late flowering, [82], and defects in ABA and glucose signaling [83], resulting in dramatic genome-wide changes in the AS of pre-mRNAs [84].

Interestingly, AS generates two *SR45* transcripts. *SR45.1* contains a 21-nucleotide sequence that is absent from *SR45.2* due to the selection of an alternative 3′ splice site in intron 6 in *SR45.1* [85,86]. These two splice variants encode similar proteins, differing by only eight amino acids, which include several putative phosphorylation sites. Remarkably, complementation studies showed that *SR45.1* rescued the floral but not the root phenotype in *sr45-1*, while *SR45.2* could complement the root growth defect observed in *sr45-1* [86]. By contrast, AS of *SR45* does not likely play a role in sugar signaling, as both splice forms were able to rescue the glucose hypersensitivity of the *sr45-1* [84].

## 5. Biotic Stress Responses

Plants are often attacked by a variety of pathogens, including fungi, viruses, and bacteria, as well as by insects and nematodes. Thus, plants have developed a number of defense mechanisms against these pathogens during evolution. Resistance (R) proteins are crucial proteins for plant defense against pathogens. The AS of *R* genes plays crucial roles in the regulation of plant defense responses at the post-transcriptional level [87,88,89].

Most *R* genes in plants encode the nucleotide-binding site (NBS) leucine-rich repeat (LRR) proteins which are characterized by containing NBS and LRR domains [90,91,92,93,94]. The coding region of most TIR-NBS-LRR genes contains three or four extrons. Among them, the first exon encodes the TIR domain, the second exon encodes the NBS domain, and the remaining exons encode the LRR region. Alternative isoforms have been observed from many TIR-NBS-LRR genes, including tobacco (*Nicotiana tabacum*) *N* and *Bs4*, *Arabidopsis RAC1*, *RPS4*, *RPS6*, *RPP5*, and *SNC1* [95,96,97,98,99,100,101]. The tobacco *N* gene confers resistance to the tobacco mosaic virus (TMV). AS produces two transcript variants: a short *NS* transcript encoding the functional N protein and a long *NL* transcript containing an alternative 70-nucleotide exon within the third intron that leads to a frame shift and PTC. Thus, the long *NL* transcript encodes a truncated protein that lacks most of the LRRs. Before infection, NS is prevalent. At 4–8 h after TMV infection, however, the level of *NL* is 60-fold higher than that of *NS*. Plants overexpressing only NS show little resistance to TMV. Thus, the alternative exon in intron 3 is required for full resistance to TMV in tobacco [102].

Similar to the tobacco *N* gene, *Arabidopsis RPS4* confers resistance to *Pseudomonas syringae* pv. *tomato* strain DC3000 (*DC3000*) expressing AvrRps4; however, this resistance is regulated by AS. Due to premature stop codons, the alternatively spliced isoforms encode no or a reduced number of LRRs. Transformation of the genomic sequence lacking intron 2 or 3 driven by the *RPS4* promoter into *rps4* revealed that the deletion any of the introns was sufficient to abolish the function of RPS4. Therefore, AS of *RPS4* is required for *Arabidopsis* to resistance to *DC3000* [99,103]. AS produces different transcript isoforms, and thus produces protein variants which may contain a combination of the TIR and NBS domains or only the TIR domain [104].

Screening for suppressors of the gain-of-function mutant suppressor of npr1-1 constitutive1 (*snc1*) in *Arabidopsis* identified a series of modifier Of *snc1* (*MOS*) genes, some of which encode subunits of a splicing-associated protein complex, including modifier Of *snc1*, 4 (MOS4), cell division cycle 5 (CDC5), and pleiotropic regulatory locus 1 (PRL1) [105]. Interestingly, their homologs in humans and yeast are components of the Prp19 complex, which is essential for catalytic activation of the spliceosome [106]. MOS4-associated complex (MAC)3A and MAC3B, two closely related proteins with sequence homology to Prp19, were identified by immunoprecipitation followed by mass spectrometry. Reported defects in the AS of *SNC1* in *mos4*, *cdc5*, and *mac3a mac3b* demonstrate that MAC mediates the AS of *R* genes and influences plant defenses [107]. MAC5A is also involved in pathogen defense [108]. The MAC5A counterpart in humans is RNA-binding motif protein 22, which interacts with U6 small nuclear RNA and pre-mRNA and participates in splicing. This suggests that MAC5A is also involved in pre-mRNA splicing in *Arabidopsis* [109]. All the results above suggest that AS is required for plants in response to biotic stresses.

## 6. Splicing Factors or Transcriptional Co-Regulators in Plants

Many splicing factors are not only components of the spliceosome that participate in pre-mRNA splicing, they can also interact with other proteins to form complexes that regulate different biological processes. Recent research has demonstrated that some plant splicing factors play an important role in transcriptional regulation.

**Figure 3:**
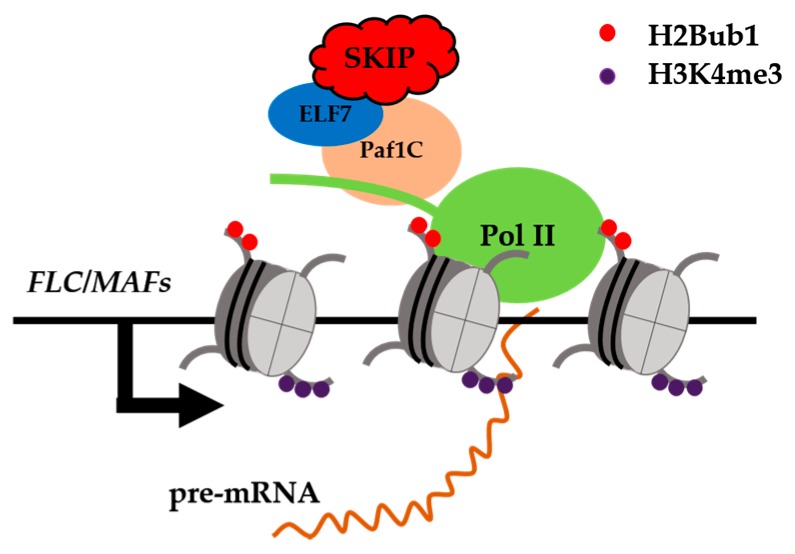
SKIP interacts directly with *EARLY FLOWERING* 7 (*ELF7*) to promote *FLC*/*MADS AFFECTING FLOWERING* (*MAFs*) transcription. SKIP is a transcriptional activator that interacts with the ELF7, a component of the Pol II associated factor 1 complex (Paf1c), to regulate the levels of H2B monoubiquitination (H2Bub1) and H3K4 trimethylation (H3K4me3) in *FLC* clade gene chromatin. SKIP represses the floral transition mainly by activating the transcription of *FLOWERING LOCUS C* (*FLC)* clade genes in *Arabidopsis*.

SKIP is a spliceosome component that interacts with the pre-mRNAs of circadian clock genes and is essential for regulating their AS [57]. Still, SKIP is not involved in the splicing of sense or anti-sense *FLC* pre-mRNA. However, the levels of mature and unspliced *FLC* mRNAs were found to be obviously repressed in *skip-1*. Yeast two-hybrid screening showed that SKIP interacts with ELF7, a component of the Paf1 complex (Paf1c), which is conserved from yeasts to humans and plants [110,111]. The Paf1c is required for the recruitment of histone modification factors and chromatin remodeling factors, and for small RNA-mediated gene silencing [112,113]. In *Arabidopsis*, the Paf1c represses the floral transition by activating *FLC* transcription [110]. The Paf1c modulates the expression of *FLC* clade genes at the transcriptional level by binding to and mediating H2B mono-ubiquitination (H2Bub1) and the tri-methylation of lysine 4 on histone H3 (H3K4me3) of *FLC* clade gene chromatin in *Arabidopsis* [111]. The results of a chromatin immunoprecipitation assay suggested that SKIP and the Paf1c are required for the H2Bub1 and H3K4me3 of *FLC* clade gene chromatin [111], supporting the proposed interaction between SKIP and the Paf1c in the regulation of flowering in *Arabidopsis.* Therefore, SKIP appears to be involved in two complexes. It can function as a splicing factor that ensures the accurate splicing of pre-mRNAs on a genome-wide scale by interacting with other components of the spliceosome, and as a transcriptional activator that interacts with other transcriptional regulators (e.g., the Paf1c) to regulate the expression of specific genes at the transcriptional level [62,111] (Figure 3). However, whether the function of these two complexes is independent or coupled through SKIP is unknown [111].

RNA-mediated transcriptional silencing is an evolutionary conserved mechanism that is required for the maintenance of genome stability, repression of transposable elements, and regulation of gene expression in eukaryotes [114,115,116]. The methylation of DNA in transposons and in other DNA repeats is conserved from plants to animals. In *A. thaliana*, an RNA-directed DNA methylation (RdDM) pathway directs de novo DNA methylation [116]. By screening for suppressors of *Repressor of Silencing 1* (*ros1*), a series of genes involved in RdDM has been identified [117,118]. Interestingly, some of these suppressors are splicing factors, including SR45 [119], ZOP1 [120], STA1 [121], PRP31 [122], and RDM16 [123].

ZOP1, an N-terminal C2H2-type ZnF and OCRE domain-containing protein, promotes DNA polymerase (Pol) IV-dependent small interfering RNA (siRNA) accumulation, DNA methylation, and transcriptional silencing. High-throughput mRNA sequencing analysis demonstrated that ZOP1 works as a pre-mRNA splicing factor, and it associates with several typical splicing machinery components. Immunofluorescence assays demonstrated that ZOP1 overlaps with the Cajal bodies and is partially co-localized with NRPE1 and DRM2 [120].

STA1 is a PRP6-like splicing factor, and is predominantly required for the accumulation of siRNAs that depend on both Pol IV and Pol V. The *sta1* mutation results in partially reduced levels of Pol V-dependent RNA transcripts. High-throughput mRNA sequencing and RT-PCR analysis demonstrated that *sta1* has no effect on the transcription of RdDM genes, suggesting that STA1 has a direct role in the RdDM pathway. Immunolocalization assays suggested that STA1 signals were exclusively present in the Cajal bodies and overlapped with that of AGO4 in most nuclei [121].

*RDM16* encodes a homolog of yeast pre-mRNA-splicing factor 3 (Prp3), a component of the U4/U6 snRNP, which is required for pre-mRNA splicing in yeast. RNA-Seq analysis showed that 308 intron retention events were observed in *rdm16* compared to that in wild-type plants, which confirmed that RDM16 is required for pre-mRNA splicing in plants. The *rdm16* mutant exhibits morphological defects and is hypersensitive to ABA signal and salt stress. However, RDM16 is likely to be directly involved in the RdDM pathway as *rdm16* does not exhibit the defection in the splicing of other RdDM genes tested [123].

**Figure 4:**
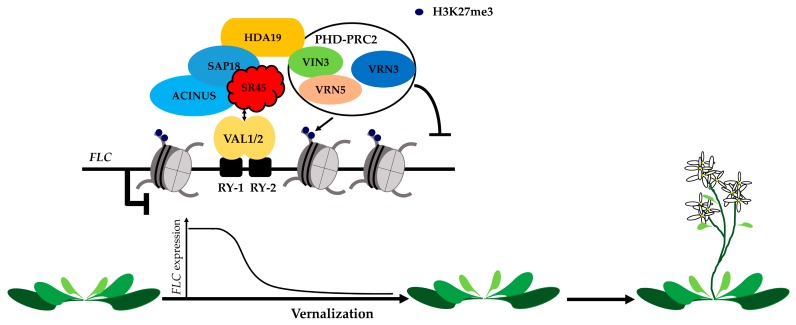
Apoptosis- and splicing-associated protein (ASAP) complex is involved in Polycomb silencing of *FLC* expression during vernalization. During vernalization, VAL1/2 bind the RY-1/2 *cis*-elements in the first intron of *FLC*, interact with components of the conserved ASAP complex, which consists of SIN3-associated protein 18 (SAP18), ACINUS and SR45, and further recruit PHD-PRC2. This transcriptional repression complex triggers histone deacetylation in *FLC* chromatin and leads to *FLC* transcriptional silencing in a sequence specific manner during vernalization. HDA: histone deacetylase. PRC2: polycomb repressive complex 2. VIN3: vernalization insensitive 3. VRN: vernalization. VAL: viviparous1/ABI3-like.

As mentioned above, SR proteins play important roles in constitutive splicing and AS, and in other aspects of mRNA metabolism. The *Arabidopsis* SR45 protein was first identified in a yeast two-hybrid screen as an interacting partner of U1-70K [124], a component of the U1 snRNP known in animals to initiate spliceosome assembly by binding 5′ pre-mRNA splice sites [125,126]. SR45 can also interact with the spliceosomal factor U2AF35, which is involved in 3′ splice site recognition [9,127]. Moreover, SR45 has been found to interact with three U5 snRNPs [128] as well as with several other *Arabidopsis* SR proteins; namely, SCL33, RSZ21, SR30, SR34, and SR34a [124,128,129]. Two other reported SR45-interacting proteins in *Arabidopsis* are the spliceosomal component SKIP [57], which confers salt stress tolerance [130], and CACTIN, an essential nuclear factor required for embryogenesis [131]. The *sr45-1* mutant exhibits delayed flowering under both long- and short-day conditions and can be rescued by vernalization. FLC, a key flowering repressor, is up-regulated in *sr45-1*. Recently, proteomics was used to identify the SIN3-associated protein 18 (SAP18) and its two partners, the RNA-binding protein SR45 and the SAP-domain protein ACINUS. SAP18 is a component of the SIN3-histone deacetylase complex, which is required in humans to enhance the SIN3-mediated repression of transcription [132]. SAP18 has also been linked to RNA processing and degradation and is a subunit of the conserved apoptosis- and splicing-associated protein complex. Together with SR45 and ACINUS, SAP18 is required for VAL1-mediated *FLC* silencing [133] (Figure 4).

Recently, genome-wide identification of RNA targets of SR45, using RNA immunoprecipitation (RIP) followed by high-throughput sequencing (RIP-seq), uncovered the unexpected roles of this RNA binding protein in RNA processing. More than 4000 RNAs are associated with SR45 directly or indirectly. Interestingly, 340 SR45-associated RNAs belong to the intronless genes. Based on the results from the genome-wide analysis, it seems that SR45 does not work as a splicing factor in regulating mRNA processing of intronless gene [134].

Therefore, some splicing factors, including SKIP, SR45, ZOP1, STA1, and RDM16, work as both a splicing factor and a transcriptional co-regulator. Whether these factors couple the transcriptional and post-transcriptional regulation of gene expression requires further investigation.

## 7. Perspectives and Conclusions

**Figure 5:**
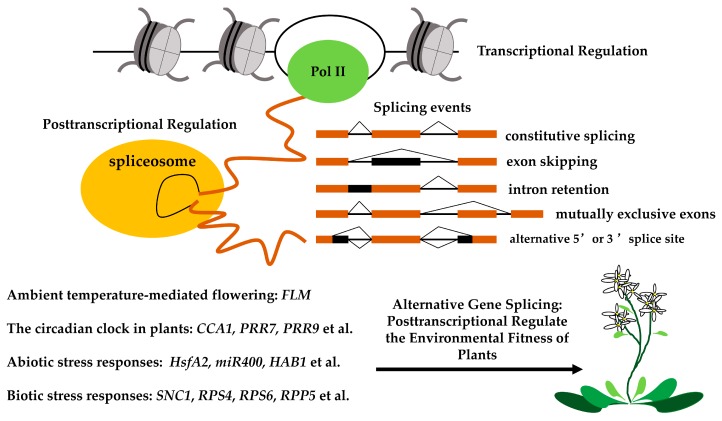
Alternative gene splicing is a novel means of regulating the environmental fitness of plants. Processing of pre-mRNA splicing is initiated during transcription. Splicing is performed by the spliceosome, a large ribonucleoprotein (RNP) complex that assembles around splice sites in the introns of a pre-mRNA molecule and then removes the introns catalytically through sequential phosphodiester transfer reactions. One pre-mRNA generate different mRNA isoforms via alternative splicing events, such as usage of an alternative 3′ or 5′ splicing site, exon skipping, intron retention, and mutually exclusive exons. Given their sessile nature, plants must adapt immediately to environmental changes to ensure their survival. Alternative splicing (AS) plays an important role in the environmental fitness of plants through alternative gene splicing.

Previous studies focused on the role of gene expression in plants in response to environmental changes at the transcriptional level. However, accumulating evidence supports the idea that the post-transcriptional regulation of gene expression plays critical roles in the response of plants to environmental changes as well. AS is a key process in post-transcriptional gene regulation; thus, it is a new means of regulating the environmental fitness of plants (Figure 5).

Thus far, analyses of AS at the whole-genome level have depended mainly on a high-throughput RNA-Seq approach. AS can enhance the complexity of the transcriptome. However, RNA-Seq data do not distinguish functional from nonfunctional isoforms, and thus may miss functional differences among splice variants.

**Figure 6:**
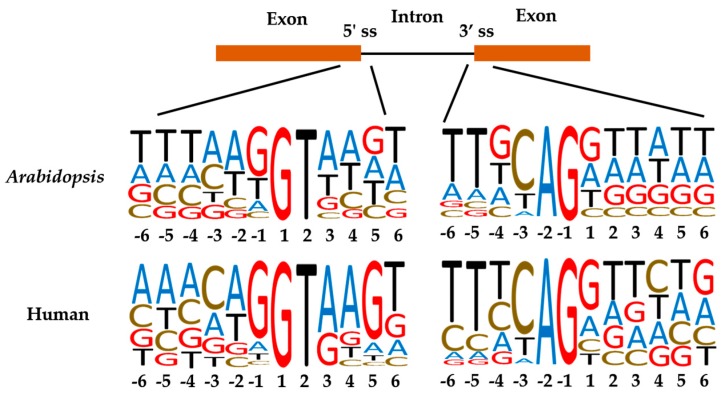
The frequency distribution of nucleotides at the 5′ splice site (5′ ss) and 3 splice site (3′ ss) in *Arabidopsis* and human. The frequencies of A, T, C, and G at each position are represented by the height of the corresponding letter.

Plant and animal introns have similar 5′ and 3′ splice sites (GU-AG). However, compared to those in animals, plant introns have their own characteristics in terms of size, nucleotide composition, branch point sequence, and polypyrimidine tract. These differences indicate that some of the initial events in splice site recognition are likely unique to plants (Figure 6) [57,135].

Some components of plant spliceosomes have been identified. Functional analyses of these spliceosomal proteins indicate that the core spliceosomal machinery to conduct splicing is evolutionary conserved between animals and plants (Table 1). However, plants have splicing proteins which do not have homologs in animals, suggesting the existence of the specific recognition of splice site and splicing regulatory mechanisms in plants (Table 1).

## Figures and Tables

**Table 1:** Plant splicing factors and their human homologs.

Plant Splicing Factor	Human Homolog	References
U1-70K	U1-70K	[60]
SKIP	SKIP	[57]
SR45	None	[124]
STIPL1	TFP11	[58]
GEMIN2	GEMIN2	[63]
SICKLE	None	[64]
MOS4	SPF27	[108]
MAC3A/3B	Prp19	[108]
CDC5	CDC5L	[108]
ZOP1	None	[120]
STA1	Prp6	[121]
PRP31	Prp31	[122]
